# Rise of the planet of rare anemias: An update on emerging treatment strategies

**DOI:** 10.3389/fmed.2022.1097426

**Published:** 2023-01-09

**Authors:** Bruno Fattizzo, Irene Motta

**Affiliations:** ^1^Fondazione IRCCS Ca’ Granda Ospedale Maggiore Policlinico, SC Ematologia, Milan, Italy; ^2^Department of Oncology and Hemato-Oncology, University of Milan, Milan, Italy; ^3^Fondazione IRCCS Ca’ Granda Ospedale Maggiore Policlinico, SC Medicina ad Indirizzo Metabolico, Milan, Italy; ^4^Department of Clinical Sciences and Community Health, University of Milan, Milan, Italy

**Keywords:** beta-thalassemia, sickle cell disease, congenital hemolytic anemias, pyruvate kinase deficiency, autoimmune hemolytic anemia, cold agglutinin disease, paroxysmal nocturnal hemoglobinuria, aplastic anemia

## Abstract

Therapeutic options for rare congenital (hemoglobinopathies, membrane and enzyme defects, congenital dyserythropoietic anemia) and acquired anemias [warm autoimmune hemolytic anemia (wAIHA), cold agglutinin disease CAD, paroxysmal nocturnal hemoglobinuria (PNH), and aplastic anemia (AA)] are rapidly expanding. The use of luspatercept, mitapivat and etavopivat in beta-thalassemia and pyruvate kinase deficiency (PKD) improves transfusion dependence, alleviating iron overload and long-term complications. Voxelotor, mitapivat, and etavopivat reduce vaso-occlusive crises in sickle cell disease (SCD). Gene therapy represents a fascinating approach, although patient selection, the toxicity of the conditioning regimens, and the possible long-term safety are still open issues. For acquired forms, wAIHA and CAD will soon benefit from targeted therapies beyond rituximab, including B-cell/plasma cell targeting agents (parsaclisib, rilzabrutinib, and isatuximab for wAIHA), complement inhibitors (pegcetacoplan and sutimlimab for CAD, ANX005 for wAIHA with complement activation), and inhibitors of extravascular hemolysis in the reticuloendothelial system (fostamatinib and FcRn inhibitors in wAIHA). PNH treatment is moving from the intravenous anti-C5 eculizumab to its long-term analog ravulizumab, and to subcutaneous and oral proximal inhibitors (anti-C3 pegcetacoplan, factor D and factor B inhibitors danicopan and iptacopan). These drugs have the potential to improve patient convenience and ameliorate residual anemia, although patient compliance becomes pivotal, and long-term safety requires further investigation. Finally, the addition of eltrombopag significantly ameliorated AA outcomes, and data regarding the alternative agent romiplostim are emerging. The accelerated evolution of treatment strategies will need further effort to identify the best candidate for each treatment in the precision medicine era.

## 1. Introduction

Rare anemias encompass several nosological entities, including inherited and acquired forms, which may present at various ages and with heterogeneous features. The former includes congenital defects of the number and structure of globin genes, as in alpha- and beta-thalassemia and sickle cell disease (SCD), as well as alterations of erythrocyte membrane or enzymes, as in congenital hemolytic anemias (CHAs). Acquired forms include immune-mediated destruction of erythrocytes [i.e., autoimmune hemolytic anemias (AIHAs)] or bone marrow precursors [i.e., aplastic anemia (AA)], and the very rare paroxysmal nocturnal hemoglobinuria (PNH). The rarity of these entities, along with the several clinical/laboratory overlaps, results in frequent misdiagnosis and delays in proper treatment. For ages, therapy of rare anemias has mainly relied on transfusion support for congenital forms and PNH, and traditional immunosuppressive treatments for acquired ones. In the last decade, a deeper understanding of physiopathology, particularly regarding the underlying molecular mechanisms, led to the development of several targeting agents. The rise of a new era of personalized medicine for rare anemia is ongoing, moving from supportive treatment to disease-modifying agents and the advent of gene therapy. This review will provide a snapshot of novel therapies for rare anemias to highlight the most recent advances in the field.

## 2. Congenital anemias

### 2.1. Update on hemoglobinopathies

**FIGURE 1:**
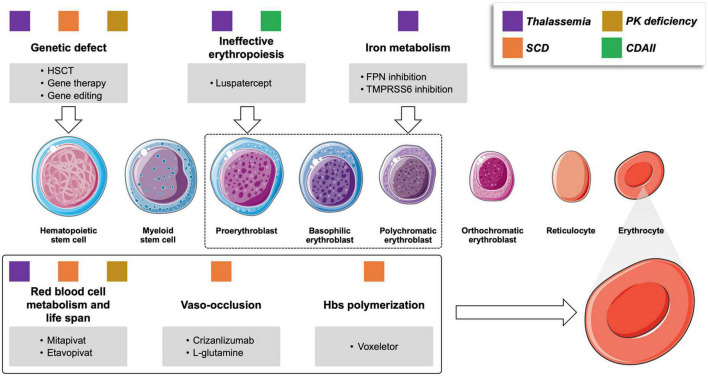
Novel drugs for rare congenital anemias and their targets. Colored squares represent the different conditions that may benefit of the various compounds under investigation. PK pyruvate kinase; CDAII congenital dyserythropoietic anemia type II; SCD sickle cell anemia; HSCT, hematopoietic stem cell transplant; PK, pyruvate kinase; FPN, ferroportin.

Hemoglobinopathies, including thalassemia and SCD, are the most common monogenic diseases worldwide (1). Although conventional supportive treatment, including transfusion programs and iron chelation therapy, has been highly optimized, these strategies still encounter significant limitations leading to morbidity and mortality. New treatment approaches and novel therapies have been proposed (Table 1), some of which have the potential to change the natural history of the disorders. Although thalassemia and SCD carry a hemoglobin (Hb) chain defect, they have different pathophysiology and clinical complications (2, 3). Emerging therapies for beta-thalassemia aim to target α/β chain imbalance, ineffective erythropoiesis, and iron dysregulation and overload. In SCD the main targets are reducing the amount of Hb S (HbS), preventing red cell dehydration or sickling, endothelial adhesion, and oxidative stress (4) (Figure 1).

**TABLE 1:** Novel drugs in patients with rare congenital anemias.

Disease	Drug	Phase/Status	Target
β-Thal	Luspatercept	FDA and EMA approved	Ineffective erythropoiesis
	Mitapivat	Phase 2	Pyruvate kinase activator
	LentiGlobin (BB305)	FDA and EMA approved	Gene therapy
	CTX001	Phase 3	Gene editing
	Vamifeport	Phase 2a	ferroportin inhibitor
	Sapablursen	Phase 2	TMPRSS6 inhibitor
SCD	Voxelotor (GBT440)	FDA and EMA approved	HbS polymerization
	Crizanlizumab	FDA and EMA approved	Vaso-occlusion
	Mitapivat	Phase 2/3	Pyruvate kinase activator
	Etavopivat	Phase 1	Pyruvate kinase activator
	L-glutamine	FDA approved/Phase 3	Substrate of NAD + synthetase
	LentiGlobin (BB305)	Phase 3	Gene therapy
	CTX001	Phase 3	Gene editing
PKD	Mitapivat	FDA and EMA approved	Pyruvate kinase activator
	RP-L301	Phase 1	Gene therapy

β-Thal, beta-thalassemia; SCD, sickle cell disease; PKD, pyruvate kinase deficiency; FDA, food and drug administration; EMA, European Medical Agency.

#### 2.1.1. Thalassemias

Luspatercept is the first-in-class erythroid maturation agent approved by the Food and Drug Administration (FDA) and European Medicine Agency (EMA) for transfusion-dependent thalassemia (TDT). Luspatercept is a recombinant fusion protein that binds ligands of the transforming growth factor beta (TGF-β) superfamily, thus inhibiting SMAD2/3 signaling and promoting late-stage erythropoiesis. The phase III BELIEVE trial showed that a significantly greater percentage of patients receiving luspatercept achieved the primary endpoint of a ≥33% reduction in transfusion burden from baseline during weeks 13–24, with a reduction of ≥2 RBC units compared with placebo (5). Data from the 5-year open-label extension phase (NCT04064060), which is currently ongoing, showed that 125 patients (55.8%) completed 144 weeks of treatment with luspatercept (6). The main reasons for treatment discontinuation were patient withdrawal (23.7% luspatercept vs. 11.6% placebo) and adverse events (10.3 vs. 1.8%), which included headache, arthralgia, bone pain, dizziness, nausea, hypertension, jaw pain, and hyperuricemia. More than 75% of patients had a reduction in transfusion burden ≥33%, and 50% had a reduction ≥50%. The transfusion window for ≥50% responders increased by 9.88 days, with 12.1% of patients achieving transfusion independence ≥8 weeks (placebo 1.8%, *P* = 0.0015). Long-term luspatercept treatment resulted in a decreasing trend in liver iron concentration compared with baseline. No new safety concerns were reported, and the occurrence of treatment-emergent adverse events of special interest was comparable with previous reports (7). Recently, the results of the BEYOND trial of luspatercept in NTDT have been published, showing that 77% of 96 patients in the luspatercept group and none in the placebo group had an increase of at least 1⋅0 g/dL of Hb. Mitapivat, initially investigated in pyruvate kinase deficiency (PKD) (see dedicated paragraph) is currently under evaluation also in alpha and beta non-transfusion-dependent thalassemia (NTDT) (NCT03692052) (8). Sixteen out of twenty (80%) patients showed an increase in Hb ≥ 1.0 g/dL, along with improvements in markers of hemolysis and ineffective erythropoiesis. Long-term data on 17 patients with a median duration of treatment of 70.9 weeks showed that Hb improvements achieved in the core period were sustained as well as improvement of markers of hemolysis and ineffective erythropoiesis. The safety profile was consistent with that observed in the core period. Headache and back pain were reported in ≥15% of patients; however, none were grade ≥3 (8). Molecules targeting iron metabolism include ferroportin inhibitor vamifeport (VIT-2763) and those up-regulating hepatic hepcidin production through inhibition of transmembrane serine protease 6 (TMPRSS6). Vamifeport improved anemia and erythropoiesis in a mouse model of β-thalassemia (9). A phase IIa double-blind, randomized, placebo-controlled study with the primary endpoint of assessing the safety and tolerability of vamifeport compared to placebo in NTDT patients ł12 years has been completed, but the results have not been published yet (NCT04364269). Antisense oligonucleotides that inhibit TMPRSS6 have shown promising results in β-thalassemia mouse models by reducing the iron burden and improving ineffective erythropoiesis (10), and a clinical trials with sapablursen is currently ongoing (NCT04059406).

#### 2.1.2. Sickle cell disease

For many years hydroxyurea has been the only pharmacological option for SCD patients, while more recently, a significant acceleration in potential treatment approaches has been observed. Indeed, EMA and FDA have recently approved two new compounds: voxelotor and crizanlizumab. Voxelotor is a Hb modulator with a good safety profile, which inhibits the polymerization of HbS stabilizing the hemoglobin in the oxygenated status. In the phase 3 trial, a significant increase in Hb levels and a decrease in markers of hemolysis were observed. However, no significant reduction in vaso occlusive crises (VOCs) was demonstrated (11). Crizanlizumab is a monoclonal antibody against P-selectin, an adhesion factor expressed by endothelium cells involved in the formation of aggregates between platelets and leukocytes, thus contributing to vessels occlusion in the microcirculation. Crizanlizumab reduced the rate of SCD–related pain crises compared to placebo, with a reduction of the annual rate of crises of 45.3% in the high-dose group (5 mg/kg). In the low-dose crizanlizumab group (2.5 mg/kg) a reduction of 32.6% compared with placebo was observed, although not statistically significant (12). Adverse events observed in at least 10% of patients in the crizanlizumab group were headache, back pain, nausea, arthralgia, pain in upper and lower limbs, urinary tract and upper respiratory infections, pyrexia, diarrhea, musculoskeletal pain, pruritus, vomiting, and chest pain. Interestingly, mitapivat is also under investigation in these patients (NCT05031780). The drug was generally well tolerated, a reduction of VOCs was observed, together with an increase in Hb levels and a decrease in markers of hemolysis (13). Etavopivat, another selective activator of erythrocyte pyruvate kinase (PKR), increases PKR activity, resulting in decreased 2,3-DPG and increased ATP. Data from the Phase 1 study (NCT03815695) showed that etavopivat 400 mg once daily was generally well tolerated. Another molecule approved only by FDA for patients older than 5 years is L-glutamine, an amino acid used by the enzyme NAD + synthetase to produce NAD + from NADH, an essential cofactor in redox reactions whose requirement is increased in SCD. A phase 3 trial showed a significant reduction in VOCs and hospital visits in children and adults treated with L-glutamine compared to placebo (14). EMA did not approve the drug for efficacy concerns.

#### 2.1.3. Gene therapy for beta-thalassemia and sickle cell disease

Allogeneic hematopoietic stem cell transplantation (HSCT) has been the only curative option for hemoglobinopathies for decades. Meanwhile, gene therapy and gene editing trials have been developed, leading to the approval in 2019 by EMA and in 2022 by FDA of the first additive gene therapy product betibeglogene autotemcel (LentiGlobin BB305) for TDT patients non-beta0/beta0. However, in March 2022, the European Commission withdrew the marketing authorization for betibeglogene autotemcel in the European Union as requested by the marketing authorization holder (bluebird bio) for commercial reasons. The same product is under investigation in SCD, with more than 30 patients treated, showing promising results (15). Of note, in 2021 a temporary suspension of the clinical trials and EMA license was announced because of a case of acute myeloid leukemia in a SCD patient treated with LentiGlobin BB305, which afterward was demonstrated as not associated with the vector (16). A gene-editing strategy that aims to reactivate HbF inhibiting BCL11A is CTX001, a CRISPR/Cas9-modified autologous HSCT product currently investigated in both TDT and SCD (17). Updated efficacy and safety data have been reported for the first 75 patients in the CLIMB THAL-111 (NCT03655678) and CLIMB SCD-121 (NCT03745287) trials, with a median follow-up of 12.3 and 9.6 months, respectively. CTX001 infusion led to the independence from transfusions in almost all patients with TDT (42/44 patients), with a sustained increase in HbF and thereby of total Hb levels (mean of >9 g/dL). All SCD patients (*n* = 31) no longer presented severe VOCs after CTX001 infusion with a mean HbF increase of ∼40% at month 4 and attainment of mean Hb levels >11 g/dL (18).

### 2.2. Update on congenital hemolytic anemias

Congenital hemolytic anemias are characterized by reduced lifespan and early destruction of erythrocytes. They encompass defects of the erythrocyte membrane proteins (hereditary spherocytosis, HS, hereditary elliptocytosis, HE, and hereditary stomatocytosis, Hst) and red cell enzymes metabolism (glucose-6-phosphate dehydrogenase, G6PD, and pyruvate kinase, PK), as well as alterations of erythrocyte precursors, resulting in defective erythropoiesis (congenital dyserythropoietic anemia, CDA) (19). Current management of CHAs mainly relies on transfusions, iron chelation, and splenectomy. The latter is highly effective in HS, less in PKD and CDA, and contraindicated in Hst due to the increased thrombotic risk. The greatest therapeutic advances for CHAs regard PKD (Table 1), including therapies that boost enzyme activity by activating PK and gene therapy (20).

#### 2.2.1. Pyruvate kinase activators

Mitapivat (AG-348), is an oral allosteric activator of erythrocyte PK. In a pivotal phase 2 study (NCT02476916) (21) enrolling 52 adults with PKD not requiring transfusions, a Hb increase of more than 1 g/dL from the baseline was reported in 26 patients (50%, mean maximum Hb increase of 3,4 g/dL, range 1.1 + 5.8) with a favorable safety profile. Hb response occurred only in patients with at least one missense mutation of the PK gene (i.e., those with residual PK activity), highlighting the importance of assessing the underlying molecular defect. Further two phase 3 trials assessed mitapivat in PKD patients requiring or not transfusion support (ACTIVATE-T and ACTIVATE) studies (NCT03548220) (22). In the ACTIVATE-T open label study (NCT03559699) (23), 37% met the primary endpoint of >33% reduction in transfusion burden, and 6 (22%) became transfusion independent. In the ACTIVATE trial of mitapivat vs. placebo, 40% achieved a sustained Hb response vs. 0 patients in the placebo arm. Data from ACTIVATE and ACTIVATE-T confirmed the long-term reduction of transfusion need in both regularly and not-regularly transfused patients (24, 25) along with Hb normalization in a proportion of patients (26). Additionally, mitapivat was shown to improve ineffective erythropoiesis and iron overload (27). Furthermore, despite mitapivat effect on aromatase, bone mineral density remains stable during long-term treatment confirming a good safety profile (28). On these bases, two novel trials with mitapivat in regularly and not-regularly transfused children with PKD have been announced (29, 30). Finally, an elegant preclinical study showed that mitapivat induced similar Hb improvement and reticulocyte decrease as splenectomy in a murine model of HS, heralding its use even in this setting (31).

#### 2.2.2. Gene therapy

In a murine model of PKD, transplantation of hematopoietic stem cells transfected with a lentiviral vector carrying PK gene restored normal glycolytic activity and erythropoiesis, and improved hemolysis. Gene therapy by lentiviral transduction of autologous stem cells and progenitor cells is under study in an open-label phase I trial (NCT04105166). Preliminary data on two adult splenectomized patients showed a Hb increase from baseline in both, along with hemolytic markers improvement. Notably, no severe adverse events were reported.

### 2.3. Summary of congenital anemias

Red blood cell transfusions have been the only therapeutic option for TDT and the severe forms of SCD. However, during the last years, the approval of luspatercept for TDT, and voxelotor and crizanlizumab for SCD, have the potential to modify the current management of these patients. Gene therapy, approved for TDT by EMA and FDA, is available only in the US for market reasons. However, promising results from gene editing trials represent a potentially curative option for beta-thalassemia and SCD. Given the complex pathophysiology of these disorders and inter-patient variability, new drugs will likely be managed with a patient-tailored approach which could include a combination of different drugs according to the individual characteristics. The management of CHAs should be individualized considering the definite diagnosis (PKD vs. HS vs. Hst, etc.), different ages, comorbidities, and frequency of complications (gallstone, hemolytic and aplastic crises, and iron overload), harnessing the need for transfusions, iron chelation, splenectomy, and cholecystectomy. Splenectomy is less effective in PKD vs. HS and contraindicated in Hst. It is discouraged during the first 6 years of age since transfusion needs may spontaneously improve, and in elderly patients for infectious and thrombotic risks. The allosteric PK stimulator mitapivat is a promising new option for PKD, both alpha and beta-thalassemia, and SCD, and possibly for HS in the next future. Among PKD patients, responses in PKD are generally observed only in patients with at least one missense mutation, whilst those with more disruptive mutations represent an unmet clinical need. Gene therapy might be a chance in these cases, but the results are still preliminary and require further investigation.

## 3. Acquired anemias

### 3.1. Update on autoimmune hemolytic anemia

**FIGURE 2:**
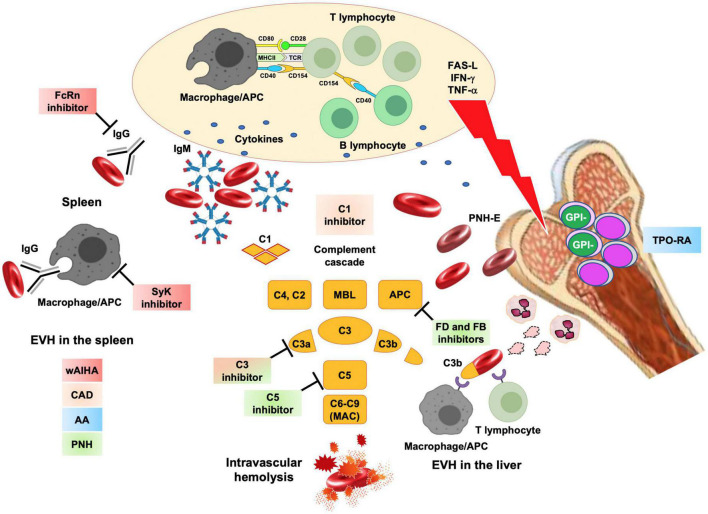
Novel drugs for rare acquired anemias and their targets. Acquired anemias encompass autoimmune hemolytic anemias, where hemolysis is due to autoantibodies produced after a tolerance break with altered B-, T- cells and antigen presenting cells (APC) crosstalk and production of several cytokines. In warm forms (wAIHA), IgG autoantibodies cause extravascular hemolysis (EVH) in the spleen. These processes may be targeted by neonatal Fc receptor inhibitors (FcRn that clear the autoantibodies from the circulation) and spleen tyrosine kinase (SyK) inhibitors (which inhibits phagocytosis). In cold agglutinin disease (CAD), IgM activate the classical complement cascade and cause C3d mediated extravascular hemolysis in the liver and minor C5 mediated intravascular hemolysis. This may be targeted by complement inhibitors (particularly C1 and C3 inhibitors). Even in wAIHA complement activation may occur and complement inhibitors are under study. Aplastic anemia (AA) is due to a T-cell attack to hematopoietic stem cells, through exposure/release of mediators such as FAS, interferon gamma (IFN) and tumor necrosis factor alpha (TNF). Thrombopoietin receptor agonists (TPO–RA) are effective, along with standard immunosuppressors, to restore hematopoiesis. After immune attack to bone marrow precursors, stem cell that acquired PIG-mutation and are glycophosphatidylinositol (GPI-) negative, may be spared and may expand in a paroxysmal nocturnal hemoglobinuria (PNH) clone. PNH erythrocytes lack natural anti-complement molecules CD55 and CD59 and are destroyed intravascularly by complement cascade (mainly through homeostatic alternative pathway activation). Along with already approved C5 inhibitors, novel drugs include C3 inhibitors, Factor B and Factor D (FB, FD). Colored squares represent the different conditions that may benefit of the various compounds under investigation. FAS-L, FAS ligand; IFN, interferon; TNF, tumor necrosis factor; macrophage/APC, antigen presenting cell; APC, alternative complement pathway.

Autoimmune hemolytic anemia is a rare disease with an incidence of 0.8 to 3/100,000 people per year and is caused by an autoimmune attack against erythrocyte antigens (32). AIHA are classified as “warm” (wAIHA) or “cold” forms (CAD), according to the thermal amplitude of the autoantibody and basing on the direct anti-globulin test (IgG + or IgG plus C3d + in wAIHA vs. C3d + and cold agglutinin detection in CAD) (32). AIHA displays multifactorial pathogenesis, including genetic (association with congenital conditions and certain mutations), environmental (drugs, infections, including SARS-CoV-2, pollution, etc.), and miscellaneous factors (solid/hematologic neoplasms, systemic autoimmune diseases, etc.) contributing to tolerance breakdown. Several mechanisms, such as autoantibody production, complement activation, monocyte/macrophage phagocytosis, and bone marrow compensation, are implicated in extra-/intra-vascular hemolysis. Management is based on standard therapies that should be differentiated and sequenced according to AIHA type. wAIHA are treated with steroids frontline, followed by rituximab, an anti-CD20 monoclonal antibody, as second line. The latter is effective in about 70–80% of cases with a median duration of response of 18 months. wAIHA patients failing rituximab represent an unmet clinical need and may be subjected to splenectomy (if young with few comorbidities) or treated with cytotoxic immunosuppressants. Frontline rituximab is advised in CAD, since steroids are effective only at high unacceptable doses. The drug induces short-term partial responses in about 50–60% of cases, and relapsed ones are handled with transfusions and cytotoxic immunosuppressants (33, 34). Novel treatments (Table 2) mainly target autoantibody production by the B-cell/plasma-cell compartment or the final erythrocyte breakdown by either complement or reticuloendothelial systems (Figure 2) (35, 36).

**TABLE 2:** Novel drugs in patients with rare, acquired anemias.

Disease	Drug	Phase/Status	Target
wAIHA	Parsaclisib	Phase 3	PI3K inhibitor
	Ibrutinib/ Rilzabrutinib	Phase 2	BTK inhibitor
	Bortezomib	Phase 2/Case reports	Proteasome inhibitor
	Daratumumab/ Isatuximab	Case reports/Phase 1	Anti-CD38 MoAb
	Pegcetacoplan	Phase 2	C3 inhibitor
	ANX005	Phase 2	Anti-C1q MoAb
	Fostamatinib	Phase 3	SyK inhibitor
	Nipocalimab/RVT-1401	Phase 3/Phase 2	Anti-FcRn MoAb
CAD	Sutimlimab	FDA and EMA approved	Anti-C1s MoAb
	Pegcetacoplan	Phase 3	C3 inhibitor
PNH	Ravulizumab	FDA and EMA approved	Anti-C5 Moab
	Crovalimab	Phase 3	Anti-C5 Moab
	Pegcetacoplan	FDA and EMA approved	C3 inhibitor
	Iptacopan	Phase 3	Factor B inhibitor
	Danicopan	Phase 3	Factor D inhibitor
	Vemircopan	Phase 2	Factor D inhibitor
	BCX9930	Phase 2	Factor D inhibitor
AA	Eltrombopag	FDA and EMA approved	TPO-RA
	Romiplostim	Phase 2/3	TPO-RA

WAIHA, warm autoimmune hemolytic anemia; CAD, cold agglutinin disease; PNH, paroxysmal nocturnal hemoglobinuria; AA, aplastic anemia; MoAb, monoclonal antibody; PI3K, phosphoinositide 3-kinase; BTK, bruton tyrosine kinase; SyK, spleen tyrosine kinase; FcRn, neonatal Fc receptor; TPO-RA, thrombopoietin receptor agonist.

#### 3.1.1. B-cell and plasma cell inhibitors

These agents include B-cell targeting agents mainly used in secondary AIHA, such as oral B-cell receptor inhibitors parsaclisib (NCT03538041 and NCT05073458), ibrutinib (NCT03827603), and rilzabrutinib (NCT05002777) that are being studied in clinical trials. In a recent multicenter, phase 2, open-label study (NCT03538041) of parsaclisib in relapsed/refractory wAIHA and CAD, the primary endpoint was the overall response at any visit from week 6 to 12. Sixteen patients (64%) responded, and 8 (32%) achieved a CR, although some toxicities emerged including diarrhea, cytomegalovirus reactivation, and psoriasis), and 2 subjects discontinued treatment (37). The drug is now being evaluated in a randomized, controlled phase 3 trial in wAIHA (NCT05073458). Among plasma cell targeting agents, the proteasome inhibitor bortezomib and the anti-CD38 MoAb daratumumab are interesting agents (38, 39). Their efficacy is supported by several case reports/series and from a single phase 2 trial of bortezomib in CAD where a 30% overall response rate was registered with limited toxicity (40). The rationale is to eliminate long-lived plasma cells that do not express CD20 and may cause rituximab refractoriness. Isatuximab, another anti-CD38 MoAb is under investigation in wAIHA in phase 1 study (NCT04661033).

#### 3.1.2. Complement inhibitors

Complement modulation is the most promising drug under study for CAD: sutimlimab, a monoclonal antibody against complement protein C1s, demonstrated a short time to response, rapid normalization of hemolysis, and good safety profile in two phase 3 trials (41, 42). Results were further updated, demonstrating long-lasting responses while on treatment (43) and reappearance of hemolysis in most patients discontinuing the drug in an 8 weeks washout periodv (44). In fact, since sutimlimab does not eliminate autoantibody production, long-term treatment is required to control hemolysis; additionally, it seems less effective on peripheral CAD-induced circulatory symptoms. Pegcetacoplan, a pegylated peptide that inhibits C3 (36), also showed good activity in a phase 2 trial in CAD and wAIHA with IgG + C DAT positivity, and a phase 3 study has been announced in CAD (NCT05096403). In wAIHA with complement activation, a novel C1q inhibitor ANX005 is also being developed (NCT04691570).

#### 3.1.3. IgG-mediated hemolysis targeting agents

The reticuloendothelial system may be targeted by inhibiting the spleen tyrosine kinase with fostamatinib, which also blocks the B-cell receptor downstream pathway (35). The drug was effective in about 45% of patients in a phase 2 trial, with mainly hypertension and diarrhea as related toxicities, and is now in phase 3 studies in wAIHA (NCT02612558). Finally, the safety/efficacy of several inhibitors of the neonatal Fc receptor (FcRn), such as intravenous nipocalimab (NCT03075878), and subcutaneous RVT-1401 (NCT04253236), are under investigation. The FcRn is structurally homologous to the MHC Class I receptor family, is expressed by several cells, and is responsible for the salvage of IgG from catabolism. Blocking FcRn induces an increased clearance of IgG, including pathogenic IgG autoantibodies.

### 3.2. Update on bone marrow failure syndromes/paroxysmal nocturnal hemoglobinuria

Bone marrow failure syndromes include the PNH–AA spectrum (45, 46). PNH is due to the acquisition of a somatic mutation of PIGA gene by the hematopoietic stem cell (HSC). PIGA encodes a glycosyl phosphatidyl inositol (GPI) molecule that anchors several factors to cell membranes. The lack of CD-55 and CD-59 GPI-anchored proteins renders PNH erythrocytes susceptible to complement-mediated destruction resulting in intravascular hemolysis with anemia and thrombosis. The expansion of PIGA-mutated HSCs to reach a clinically significant clone size is thought to be partly due to an immune attack against PNH-negative HSCs. This T-cell-mediated autoimmune attack against BM precursors is typical of AA, which is associated with PNH in up to 60% of cases (47). Up to the early 2000s, PNH therapy was mainly supportive, and AA patients received immunosuppressive treatment with anti-thymocyte globulin and cyclosporine, or, if candidate, HSC transplant, with heterogeneous and mainly age-related efficacy. In the last 15 years, the treatment of PNH and AA has been revolutionized by the introduction of complement inhibitors in the former, and of the thrombopoietin receptor agonist eltrombopag in the latter (Table 2).

#### 3.2.1. Complement inhibitors for paroxysmal nocturnal hemoglobinuria

The anti-C5 MoAb eculizumab was the first drug to reduce hemolysis, improve anemia, and abate thrombotic risk in PNH patients. The risk of Neisseria meningitidis infections mandated the vaccination with anti-Meningococcus ACYW135 and B before starting therapy, along with life-long education and monitoring of infectious risk. Additionally, the drug required fortnightly intravenous infusions, and up to 2/3 of cases had residual anemia due to persistent intravascular hemolysis, concomitant bone marrow failure, and development of extravascular hemolysis driven by C3 deposition on PNH erythrocytes (48, 49). In the last decade, the long-half-life anti-C5 ravulizumab has been studied and shown not inferior to eculizumab in two phase 3 trials in PNH naïve or previously exposed to eculizumab (50, 51) and was recently approved. Administered every 8 weeks, the drug has the potential to stabilize hematologic response and better control breakthrough hemolytic episodes. Another promising anti-C5, currently in phase 3 investigation, is crovalimab (52). It is administered subcutaneously every 4 weeks, is well tolerated, and has a different target from ecu/ravu, thus being active on the Asian C5 polymorphism. The development of drug-target-drug immune-complexes should be surveilled during the switch from ecu/ravu to crovalimab, since it may cause immunologic reactions that tend to resolve over time (53). Pegcetacoplan, previously mentioned for CAD, is a C3 inhibitor that reduced C3-mediated extravascular hemolysis and alleviated anemia and transfusion dependence in PNH patients who were suboptimal responders to eculizumab (54). The drug is infused subcutaneously twice a week and is now approved for the frontline treatment of PNH patients in the US and those anemics after at least 3 months of anti-C5 treatment in Europe. More recently, several updates on the long-term safety and efficacy of pegcetacoplan have been presented, also highlighting an anti-thrombotic effect of the drug (55–58). Oral agents targeting factor B and D of the alternative pathway represent a further innovation. Factor B inhibitor iptacopan, and factor D inhibitor danicopan have been shown to improve anemia and reduce hemolysis and transfusion needs in PNH patients who are suboptimal responders to anti-C5 in early phase trials (59, 60). The first is being developed as a single agent BID oral therapy, and a phase 3 trial is ongoing (NCT04558918); the second is a TID oral therapy given as add on to anti-C5. A more potent anti-D, vemircopan, administered as monotherapy once a day, is under study in naïve and suboptimal responders to C5 inhibition (NCT04170023). Another oral factor D inhibitor, BCX9930 is also being studied in naïve and previously treated PNH patients, and preliminary results are encouraging (NCT05116787 and NCT05116774). Notably, proximal inhibitors require vaccination with anti-Meningococcus, anti-Pneumococcus, and anti-Haemophilus before treatment start.

#### 3.2.2. Eltrombopag in aplastic anemia

Ten years ago, the NIH group published the first results regarding eltrombopag efficacy in up to 40% of AA patients relapsed/refractory to immunosuppressive treatment (61). Since then, several real-world series confirmed the use of eltrombopag as a single agent at 150 mg day in this setting with trilinear improvement in some cases (62). The addition of eltrombopag to first-line immunosuppression further improved responses to >90% in more recent reports (63) and was superior to IST alone in a phase 3 randomized European trial (64), without increasing toxicity nor clonal evolution. Treatment schedules, particularly regarding the length of eltrombopag administration, the possibility of tapering and discontinuing the drug and to re-start it in case of relapse, deserve further investigation. The drug interferes with cation-containing foods, thus requiring fasting before and after administration. Asian groups are exploring the use of the alternative TPO-RA agent romiplostim. Preliminary results seem promising, with more than 80% response rates if used frontline in association with IST (65–67).

### 3.3. Summary of acquired anemias

Novel agents represent a basket of opportunities for wAIHA and CAD, while a gray zone of uncertainness remains for treating mixed and atypical AIHA forms. B-cell targeting small molecules and anti-plasma cell agents are promising, although response rates are still lower than those obtained with rituximab, and toxicities may be higher, deserving further investigation. The spleen tyrosine kinase inhibitor fostamatinib blocks phagocytosis by the reticuloendothelial system in wAIHA, and also modulates B-cell receptor activity, potentially reducing autoantibody production. Regarding complement inhibitors, they have high efficacy in CAD; they do not eliminate the autoantibody and should likely be administered indefinitely. Similarly, anti-FcRn agents increase the clearance of pathogenic autoantibodies in wAIHA, but autoantibody production is preserved, suggesting the need for combination therapy in the future. Additionally, the very short response time to these agents may be particularly helpful in severely anemic patients and acute crises.

Regarding PNH, its treatment is facing an era of expanding options with different routes of administration. The latter will likely improve patient convenience but also pose warnings on compliance (68). Proximal inhibitors (C3, factor B and D inhibitors) show dramatic efficacy on extravascular hemolysis and in improving residual anemia while on anti-C5 agents. The “sparing” of a large PNH cell clone with these agents may, in turn, fuel severe hemolytic breakthroughs in case of complement-activating events such as infections, traumas, surgery, etc. The proper management of such pharmacodynamics breakthrough hemolysis is still unknown and will require further investigation. Other open issues include the efficacy of these novel agents in preventing thrombotic episodes, and their long-term safety, particularly regarding infections. While eculizumab has been proven safe and is indicated in the case of pregnancy, no data are available for novel agents. Finally, the introduction of eltrombopag improved the treatment of AA, particularly in the setting of relapse/refractory patients and in the elderly, given the good safety profile and the absence of kidney toxicity. Its use frontline is supported by convincing evidence, but it is still not licensed in Europe, and the timing of administration and the possibility of clonal evolution deserves further investigation. From a patient perspective, the interference with cations containing food should be considered, and future strategies, including romiplostim, and possibly the new TPO-RA agent avatrombopag that has no food interference, require further exploration.

## 4. Red blood cell transfusions in the current era

Supportive treatment with RBC transfusions remains the mainstay for the management of anemia in the acute setting of both congenital and acquired forms, as well as chronically in patients with TDT. The thresholds of Hb levels are highly heterogeneous across centers and should be carefully weighed on patient age, comorbidities, and disease type. For instance, during infancy, patients with TDT require chronic support to allow development and to avoid extramedullary erythropoiesis and skeletal deformities, whilst in CHAs, transfusions are seldom required on a regular basis. Furthermore, in PKD, the augmented levels of 2,3 diphosphoglyceric acid increase oxygen release to the tissues, thus improving anemia tolerance (19, 20). The relevant issue of alloimmunization should be considered both in poly transfused patients with congenital forms and in those with acquired autoimmune ones, where the risk is higher due to disease-related immune dysfunction (32). In the current era, the advances in phenotyping and genotyping of patients and blood donors have markedly improved unit matching, thus abating alloimmunization and transfusion reactions.

## 5. Conclusion

Therapeutic options for rare anemias are rapidly expanding and continue to ameliorate disease outcomes with reduction of transfusion need, VOCs, and iron overload in hemoglobinopathies and CHAs and improvement of hemolysis and anemia in AIHA, PNH, and AA. Interestingly, compounds designed for a specific disorder have been considered beneficial also for other anemias in a sort of repurposing process with potentially lower overall development costs and shorter development timelines. Importantly, these compounds may also improve patient convenience. On the other hand, the accelerated evolution of treatment strategies will need a further effort to identify the best candidate for each treatment in the precision medicine era. Non-responders to novel therapies are often disregarded in clinical trials and predictors of response are only seldomly explored (i.e., presence of disruptive genotype in PKD). They represent an unmet need for further development in this area. Finally, as more and more agents become available, costs are also rising for the national health systems and would require careful consideration within regulatory and clinical communities.

## Author contributions

Both authors equally contributed to the conceiving, writing, revision of the manuscript, and approved the submitted version.
